# Exercise is inversely associated with functional dyspepsia among a sample of Chinese male armed police recruits

**DOI:** 10.1186/s12876-023-03072-z

**Published:** 2023-12-08

**Authors:** Zhongcao Wei, Yan Yang, Ting Du, Yujie Hao, Na Liu, Yong Gu, Jinhai Wang

**Affiliations:** 1 Department of Gastroenterology, The Second Affiliated Hospital, Xi’an Jiaotong University, Shaanxi, China; 2 State Key Laboratory of Cancer Biology, National Clinical Research Center for Digestive Diseases and Xijing Hospital of Digestive Diseases, Air Force Medical University, 127 Changle West Road, Xi’an, 710032 Shaanxi China; 3 Digestive partment, Shaanxi Provincial Crops Hospital, Chinese People’s Armed Police Forces, Xi’an, China; 4 Department of Gastroenterology, Hainan General Hospital (Hainan Affifiliated Hospital of Hainan Medical University), Haikou, China

**Keywords:** Functional dyspepsia, Exercise, Rome IV criteria, Recruits

## Abstract

**Background:**

There is no study evaluating the association between exercise and functional dyspepsia (FD) based on the Rome IV criteria. We aimed to investigate the prevalence of FD and evaluate the association between exercise and FD based on Rome IV criteria among a sample of Chinese armed police recruits.

**Methods:**

An on-site questionnaire survey on FD among a sample of Chinese armed police recruits was conducted based on the Rome IV criteria in 2021. Potential confounders included age, body mass index (BMI), race, marriage, education, smoking, and drinking variables were adjusted.

**Results:**

A total of 2594 recruits were enrolled, including 46 FD participants and 2548 non-FD participants. In the model adjusted for all demographic variables among participants excluding irritable bowel syndrome (IBS) and functional constipation (FC), compared with no exercise participants, 1 h < each exercise time ≤ 2 h (OR = 0.15, 95% CI: 0.03–0.77, P = 0.0230) was inversely associated with FD and compared with no exercise participants, mild exercise (OR = 0.09, 95% CI: 0.01–0.71, P = 0.0220) was significantly inversely associated with FD.

**Conclusions:**

The incidence rate of FD in this sample Chinese armed police recruits was 1.77%, and 1 h < each exercise time ≤ 2 h and mild intensity exercise were independently inversely associated with FD. However, the causal relationship needs to be verified by further randomized controlled trials.

## Background

Functional dyspepsia (FD) was one of the most common functional gastrointestinal diseases. Patients mainly showed gastroduodenal symptoms such as epigastric pain, fullness and heartburn, and there are no organic or metabolic diseases that can explain these symptoms [1, 2]. A meta-analysis of the global prevalence of dyspepsia showed that the prevalence of dyspepsia was approximately 20% in the global general population. The endoscopic results of 80% of these dyspepsia patients could not explain the symptoms [3]. FD often occurred chronically and repeatedly, which seriously affects the daily work and life of patients, and even their mental health [4, 5]. And due to repeated medical treatment, it also causes huge economic losses [6, 7]. Therefore, it is particularly important to identify patients with FD as soon as possible and carry out effective intervention to improve the efficacy and strengthen the prevention of FD.

The pathogenesis of FD was still unclear, which may be related to abnormal gastrointestinal motility, dysfunction of brain-gut axis, psychosocial factors and other factors [8–10]. Studies had shown that exercise can promote gastrointestinal peristalsis and improve psychosocial factors, and exercise may have a potential therapeutic effect on FD [11]. Few studies had assessed the association between exercise and FD. A mail survey of 3160 Australians by Talley NJ et al. showed that FD was associated with lower exercise levels [12]. An online questionnaire survey of 15,000 adults in Japan showed that the exercise frequency of patients with FD was significantly lower than that of patients with non-FD [13]. Both of these studies collected data from online surveys, which may have a selection bias. A cross-sectional study published by Furukawa et al. in 2021 showed that in Japanese college students, the frequency and intensity of exercise may be inversely associated with the occurrence of FD, but there was no limit on the frequency of FD symptoms in this study [14]. And all these studies were based on the Rome III criteria, not the latest Rome IV criteria, and these observations need to be confirmed in the Chinese population.

There were few studies on functional gastrointestinal diseases in soldiers. As the fresh blood of the army, it was of great significance to explored the relationship between FD and exercise, which can provide a basis for the treatment of FD in Chinese armed police recruits. Moreover, the compliance of the recruits was good, and the questionnaire data had good authenticity and reliability, the research subjects had performed a certain amount of exercise required for this study. In this study, we aimed to evaluate the association between exercise and FD based on Rome IV criteria in Chinese armed police recruits.

## Methods

### Study population

In 2021, we conducted an on-site questionnaire survey to evaluate the association between exercise and FD based on Rome IV criteria in Chinese armed police recruits. The Armed Police Force was responsible for important tasks such as border patrol, on-duty security, maintaining stability and dealing with emergencies, fire rescue and other important tasks, and was the main force to maintain domestic order and stability in peacetime. The gastroenterologist from the Xi’an Armed Police Hospital and the Second Affiliated Hospital of Xi’an Jiaotong University distributed questionnaires to recruits, and informed them that the questionnaires were designed to investigate the occurrence of FD in recruits and were mainly used for scientific research without revealing personal information. The gastroenterologist explained the problems existing in the process of filling in the questionnaires, and the questionnaires were returned after completion. All recruits signed written informed consent before conducting the questionnaire survey. And this study was approved by the Ethics Committee of the Second Affiliated Hospital of Xi’an Jiaotong University (approval number: 2,020,031 and approval date: 2020.7.10).

### Sample size calculation

The sample size was calculated based on the incidence rate of FD. A systematic review of FD defined according to Rome criteria shows that the incidence of FD in adults worldwide is 1.8–57%. We selected the lower incidence rate of 3% as the reference standard, with an error margin of 1% and a confidence level of 95%. The PASS 15.0 software was used, and the final sample size was 1223.

### Inclusion criteria and exclusion criteria

The inclusion criteria was age ≥ 18 years. The exclusion criteria were as follows: there were organic diseases that may explain the symptoms, such as peptic ulcer, gastrointestinal tumors, history of hepatobiliary and pancreatic diseases, history of metabolic diseases, history of abdominal surgery; recent use of anti-anxiety and anti-depression drugs; incomplete data.

### Data collection

We collected the demographic data, including name, age, gender, body mass index (BMI), race, marriage, education, smoking, drinking, dyspeptic information (dyspeptic symptoms, duration, frequency and severity), daily exercise information before enlistment (number of days of exercise per week, time of each exercise, and exercise intensity).

### Definitions of FD

In this study, we used the Rome IV diagnostic criteria for FD. FD was defined as epigastric pain or epigastric burning that occurred for at least 1 day per week, or postprandial fullness, early satiety that occurred for at least 3 days per week. And the duration of dyspepsia symptoms ≥ 6 months, and the symptoms of dyspepsia in the last 3 months meet the above criteria. The diagnosis of irritable bowel syndrome (IBS) and functional constipation (FC) was also based on the Rome IV criteria [15].

### Exercise intensity classification

Exercise intensity was classified according to ratings of perceived exertion (RPE) [16]. RPE was divided into 6–20 grades according to self-perception, among which still relaxed (RPE11-12) and slightly tired (RPE13-14) correspond to moderate intensity exercise [17]. Therefore, quiet (RPE6), extremely relaxed (RPE7-8), very relaxed (RPE9-10) correspond to mild intensity exercise; tired (RPE15-16), very tired (RPE17-18), extremely tired (RPE19-20), exhaustion (RPE20) corresponds to high intensity exercise.

### Statistical analysis

EmpowerStats and SPSS20.0 software were used for data processing. The classified variables were expressed as counts and percentages, and chi-square test or fisher exact test was used. The continuous variables were expressed by mean ± standard deviation and analyzed by t-test or Kruskal-Wallis test. Using Multiple logistic regression analyses, the ability of exercise to distinguish FD from non-FD was assessed by adjusting age, BMI, race, marriage, education, smoking, and drinking variables, which had been shown in previous studies to be potentially associated with FD [18–20]. FD, IBS and FC all belong to functional gastrointestinal diseases. In order to exclude confounding, as studied by Koloski et al., we excluded two other functional gastrointestinal diseases (IBS and FC) with relatively high incidence rate, making the results of the article more atable [21]. The incidence rate of functional diarrhea was relatively low, so we did not analyze it. We have added this to the article. P < 0.05 was considered to be statistically significant.

## Results

### Baseline of participant characteristics

**Fig. 1:**
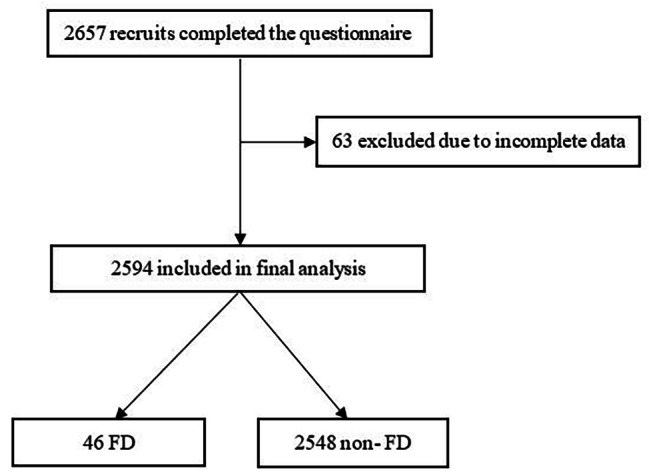
Flowchart of the study. FD: Functional dyspepsia

In our study, a total of 2657 recruits of this sample completed the questionnaire, after excluded 63 recruits due to incomplete data, there were 2594 recruits met inclusion and exclusion criteria, and included in final analysis (Fig. 1). The participants were all male, and the mean age was 19.8 ± 1.5 years, the mean BMI was 19.6 ± 2.6 kg/m^2^. And the Han nationality accounted for 98% (2544/2594), and the college level accounted for 70.3% (1823/2594). The average number of exercise days per week was 4.8 ± 1.8 days. The proportion of 1 h < each exercise time ≤ 2 h was the highest, and the proportion of moderate intensity exercise was the highest.

### Prevalence of FD, IBS and FC in recruits

**Fig. 2:**
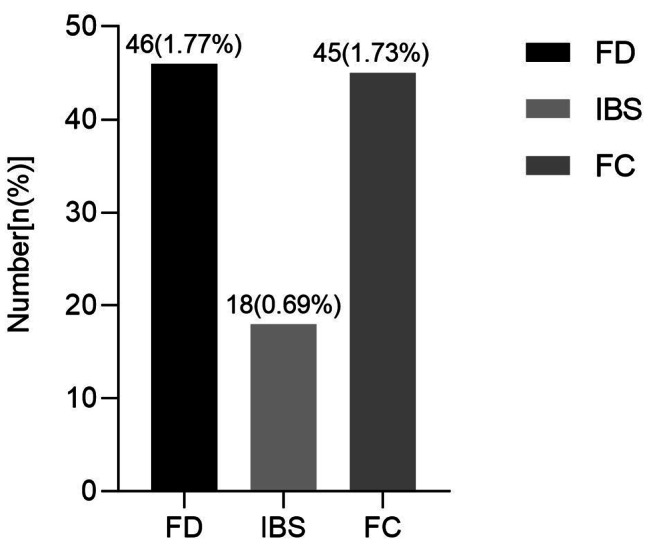
The prevalence of FD, IBS and FC in recruits. FC: Functional constipation, FD: Functional dyspepsia, IBS: Irritable bowel syndrome

Among the participants, there were 46 FD participants and 2548 non-FD participants. The incidence of FD among recruits was 1.77% (46/2594). FD participants included 14 participants with epigastric pain syndrome, 38 participants with postprandial discomfort syndrome and 6 participants with overlap syndrome. There were 18 participants met Rome IV criteria for IBS, the incidence of IBS was 0.69% (18/2594) and there were 45 participants met Rome IV criteria for FC, the incidence of FC was 1.73% (45/2594) (Fig. 2).

### Univariate association between FD and exercise

Univariate analysis showed that there were significant differences in drinking, time of each exercise and exercise intensity between the FD and non-FD participants (P < 0.05). And there was no significant difference between the FD and non-FD participants in age, BMI, race, marriage, education, smoking and exercise days per week (P>0.05). The results of univariate analysis of FD and non-FD in recruits were shown in Table 1.

**Table 1:** Univariate analysis of FD and non-FD in recruits

	All participants(N = 2594)	non-FD (N = 2548)	FD (N = 46)	*P* value
Age, years	19.9 ± 1.5	19.9 ± 1.5	19.9 ± 1.6	0.712
BMI, Kg/m^2^	19.6 ± 2.6	19.6 ± 2.6	20.0 ± 2.6	0.228
Race				0.337
Han	2544 (98.0%)	2498 (98.0%)	46 (100.0%)	
Minority nationality	50 (2.0%)	50 (2.0%)	0 (0%)	
Marriage				1
Never married	2588 (99.8%)	2542 (99.8%)	46 (100.0%)	
Married	6 (0.2%)	6 (0.2%)	0 (0%)	
Education				0.464
Junior high school and below	11 (0.4%)	11 (0.4%)	0 (0%)	
High school	760 (29.3%)	750 (29.4%)	10 (21.7%)	
University	1823 (70.3%)	1787 (70.2%)	36 (78.3%)	
Smoking	539 (20.8%)	526 (20.6%)	13 (28.3%)	0.207
Drinking	163 (6.3%)	152 (6.0%)	11 (23.9%)	<0.001
Exercise days per week, days	4.8 ± 1.8	4.8 ± 1.8	4.3 ± 2.5	0.256
Time of each exercise				<0.001
0	36 (1.4%)	31 (1.2%)	5 (10.9%)	
0< ≤0.5 h	93 (3.6%)	90 (3.5%)	3 (6.5%)	
0.5 h< ≤1 h	664 (25.6%)	650 (25.5%)	14 (30.4%)	
1 h< ≤2 h	1088 (41.9%)	1078 (42.3%)	10 (21.7%)	
> 2 h	713 (27.5%)	699 (27.4%)	14 (30.4%)	
Exercise intensity				<0.001
no exercise	36 (1.4%)	31 (1.2%)	5 (10.9%)	
Mild intensity	454 (17.5%)	452 (17.7%)	2 (4.3%)	
Moderate intensity	1538 (59.3%)	1514 (59.4%)	24 (52.2%)	
High intensity	566 (21.8%)	551 (21.6%)	15 (32.6%)	

Values are expressed as the mean ± standard deviation or n (%)

### Multivariate model analysis

In order to further explore the association between exercise and FD, we used a multivariable model for analysis. The analysis results showed that in the model without adjustment, the number of exercise days per week was inversely associated with FD, and the difference was statistically significant (P < 0.05). Compared with no exercise participants, each exercise time ≤ 0.5 h, 0.5 h < each exercise time ≤ 1 h, 1 h < each exercise time ≤ 2 h and more than 2 h were inversely associated with FD (P < 0.05). Compared with no exercise participants, mild, moderate and high intensity exercise were significantly inversely associated with FD (P < 0.05) (Table 2).

**Table 2:** Multivariate model analysis of exercise and FD

	Model 1 OR (95% CI), *P* value	Model 2 OR (95% CI), *P* value	Model 3 OR (95% CI), *P* value	Model 4 OR (95% CI), *P* value	Model 5 OR (95% CI), *P* value
Exercise days per week, days	0.85 (0.74, 0.99), *P* = 0.0397	0.91 (0.78, 1.07), *P* = 0.2416	0.90 (0.76, 1.07), *P* = 0.2277	0.93 (0.79, 1.10), *P* = 0.4200	0.93 (0.78, 1.12), *P* = 0.4381
Each exercise time					
0	1.0	1.0	1.0	1.0	1.0
0< ≤0.5 h	0.21 (0.05, 0.92), *P* = 0.0379	0.26 (0.06, 1.22), *P* = 0.0886	0.24 (0.04, 1.42), *P* = 0.1155	0.41 (0.08, 2.22), *P* = 0.3025	0.45 (0.06, 3.43), *P* = 0.4391
0.5 h< ≤1 h	0.13 (0.05, 0.39), *P* = 0.0003	0.18 (0.06, 0.55), *P* = 0.0027	0.19 (0.05, 0.66), *P* = 0.0088	0.27 (0.07, 1.05), *P* = 0.0581	0.36 (0.07, 1.75), *P* = 0.2048
1 h< ≤2 h	0.06 (0.02, 0.18), *P*<0.0001	0.08 (0.02, 0.26), *P*<0.0001	0.08 (0.02, 0.30), *P* = 0.0002	0.12 (0.03, 0.49), *P* = 0.0028	0.15 (0.03, 0.77), *P* = 0.0230
> 2 h	0.12 (0.04, 0.37), *P* = 0.0002	0.19 (0.06, 0.61), *P* = 0.0050	0.20 (0.06, 0.73), *P* = 0.0149	0.26 (0.07, 1.03), *P* = 0.0553	0.34 (0.07, 1.70), *P* = 0.1889
Exercise intensity					
no exercise	1.0	1.0	1.0	1.0	1.0
Mild intensity	0.03 (0.01, 0.15), *P*<0.0001	0.04 (0.01, 0.22), *P* = 0.0002	0.05 (0.01, 0.30), *P* = 0.0011	0.06 (0.01, 0.39), *P* = 0.0031	0.09 (0.01, 0.71), *P* = 0.0220
Moderate intensity	0.10 (0.04, 0.27), *P*<0.0001	0.13 (0.05, 0.40), *P* = 0.0003	0.15 (0.05, 0.51), *P* = 0.0021	0.21 (0.06, 0.75), *P* = 0.0166	0.29 (0.06, 1.34), *P* = 0.1129
High intensity	0.17 (0.06, 0.49), *P* = 0.0012	0.22 (0.07, 0.69), *P* = 0.0091	0.18 (0.05, 0.66), *P* = 0.0094	0.33 (0.09, 1.24), *P* = 0.0995	0.32 (0.06, 1.59), *P* = 0.1614

Model 1: All participants (n = 2594), unadjusted variables; Model 2: All participants (n = 2594), adjusted age, BMI, race, marriage, education, smoking, drinking variables. Model 3: Participants excluding IBS (n = 2576), adjusted age, BMI, race, marriage, education, smoking, drinking variables. Model 4: Participants excluding FC (n = 2549), adjusted age, BMI, race, marriage, education, smoking, drinking variables. Model 5: Participants excluding IBS and FC (n = 2531), adjusted age, BMI, race, marriage, education, smoking, drinking variables

At the same time, the multivariate model analysis showed that in the model adjusted for all demographic variables among all participants and participants excluding IBS, compared with no exercise participants, 0.5 h < each exercise time ≤ 1 h, 1 h < each exercise time ≤ 2 h, and more than 2 h were inversely associated with FD (P < 0.05); compared with no exercise participants, mild exercise, moderate exercise, and high intensity exercise were significantly inversely associated with FD (P < 0.05). And in the model adjusted for all demographic variables among participants excluding FC, compared with no exercise participants, 1 h < each exercise time ≤ 2 h was inversely associated with FD and compared with no exercise participants, mild exercise and moderate exercise were significantly inversely associated with FD (P < 0.05). Finally, in the model adjusted for all demographic variables among participants excluding IBS and FC, compared with no exercise participants, 1 h < each exercise time ≤ 2 h (OR = 0.15, 95% CI: 0.03–0.77, P = 0.0230) was inversely associated with FD and compared with no exercise participants, mild exercise (OR = 0.09, 95% CI: 0.01–0.71, P = 0.0220) was significantly inversely associated with FD. The multivariate model analysis of exercise and FD in recruits was shown in Table 2.

## Discussion

To our knowledge, our study was the first to research the association between FD and exercise in recruits. At the same time, we have adopted the latest Rome IV criteria. In this cross-sectional study, we found that in the model adjusted for all demographic variables among participants excluding IBS and FC, 1 h < each exercise time ≤ 2 h and mild intensity exercise were independently inversely associated with FD. Our study evaluated the incidence of FD in Chinese armed police recruits and explored the relationship between FD and exercise, which can provide a basis for the treatment of FD in Chinese armed police recruits.

Compared with the Rome III criteria, the Rome IV criteria redefines the frequency and severity of dyspeptic symptoms, but more studies are needed to confirm the effectiveness of the Rome IV criteria [22]. In this study, we adopted Rome IV criteria for the diagnosis of FD, the incidence of FD in recruits was 1.77%. A systematic review of FD defined according to Rome criteria shows that the incidence of FD in adults worldwide is 1.8–57% [3]. In this study, the incidence of FD was similar to the lower limit of this range. At the same time, the incidence of other functional gastrointestinal diseases such as IBS and FC in the study was also relatively low, which may be due to the relatively strict Rome IV criteria adopted in our study and the particularity of recruits. The average age of recruits in this study was 19.8 ± 1.5 years old, and the incidence rate of FD in this age group was low.

There are many studies on the influence of exercise on irritable bowel syndrome, and exercise has shown the effect of improving IBS [23, 24]. Although some gastroenterologists recommend physical activity as a first-line treatment for FD [25], there are few studies on the association between exercise and FD. FD and IBS are both functional gastrointestinal diseases [26]. And brain-gut axis dysfunction is one of the common pathogenesis of FD and IBS, exercise has shown the effect of improving IBS. Therefore, the effect of exercise on FD is theoretically feasible. In our study, we found that in the model adjusted for all demographic variables among participants excluding IBS and FC, 1 h < each exercise time ≤ 2 h and mild intensity exercise were independently inversely associated with FD. A recent small sample randomized controlled study by Rane et al. found that 6 weeks of aerobic exercise as an adjuvant therapy had a certain effect on FD [27]. Koloski et al. also showed that FD patients reported significantly less walking [21]. Therefore, we speculate that exercise may have some value in the treatment of FD. At the same time, we found that in the model 3 and model 4, 1 h < each exercise time ≤ 2 h, moderate intensity exercise and high intensity exercise were negatively associated with FD, and in model 3, each exercise time > 2 h was negatively associated with FD, while in model 5, these factors were not significantly associated with FD, the research results were unstable. Therefore, the association between each exercise time > 1 h, exercise intensity and FD still needs to be confirmed by further studies.

The underlying mechanisms of the association between exercise and FD were unclear. Studies had shown that exercise can reduce inflammatory cytokines and promote intestinal gas excretion. The study by Dainese et al. on intestinal gas transport in healthy adults showed that intestinal gas retention was significantly lower during exercise than during rest, and abdominal bloating was significantly relieved after exercise. In healthy people, mild exercise can promote gas delivery in the intestinal cavity [28]. Physical exercise may improve the symptoms of FD by reducing gastric acid reflux, increasing intestinal gas transport and inhibiting inflammatory cytokines. In our study, FD was associated with lower levels of exercise. Research by Cordner et al. showed that FD patients had a higher proportion of anxiety and depression. Therefore, anxiety and depression may be a potential cause of lower exercise levels in FD participants, but research was needed to confirm [29].

Recent study had also shown that intestinal flora disorders play an important role in the pathogenesis of FD. Changes in intestinal motility, increased intestinal permeability and visceral hypersensitivity were all related to the intestinal flora disorders [30]. Exercise may improve the symptoms of functional dyspepsia by changing the composition of intestinal microflora. Psychosocial factors were one of the pathogenesis of FD [9, 10]. Endorphins were released by the brain during physical exercise, which could help relieve stress and had a positive effect on mood [31–33]. The possible mechanism of exercise on FD needs to be further studied.

There were several limitations in our study. First of all, this study was a cross-sectional study, which can only draw the conclusion that there was a inversely association between exercise and FD, but can not draw the causal relationship between exercise and FD, which needed to be verified by further randomized controlled studies. Secondly, the recruits were special social groups, and the recruits we included in the study were all male, which may affect the extrapolation of the research results. And due to the confidentiality principle of military information, we were unable to provide more specific information on research coverage. In the future, more extensive group studies were needed to evaluate the association between exercise and FD in the general population. Thirdly, due to the impact of the COVID-19, our data collection had been hindered. The data of a small number of recruits had not been collected, but it was negligible compared to the overall data.

## Conclusions

Our study found that the incidence rate of FD in this sample Chinese armed police recruits was 1.77%, and 1 h < each exercise time ≤ 2 h and mild intensity exercise were independently inversely associated with FD. However, the causal relationship between exercise and FD still needs further randomized controlled study to verify.

## Data Availability

The datasets used and/or analyzed during the current study are available from the corresponding author on reasonable request.

## Abbreviations

FC: Functional constipation
FD: Functional dyspepsia
IBS: Irritable bowel syndrome
RPE: Ratings of perceived exertion
